# Draft Genome Sequence of the Plant Growth–Promoting *Pseudomonas* *punonensis* Strain D1-6 Isolated from the Desert Plant *Erodium hirtum* in Jordan

**DOI:** 10.1128/genomeA.01437-16

**Published:** 2017-01-12

**Authors:** Feras F. Lafi, Maha L. AlBladi, Nida M. Salem, Luma Al-Banna, Intikhab Alam, Vladimir B. Bajic, Heribert Hirt, Maged M. Saad

**Affiliations:** a King Abdullah University of Science and Technology (KAUST), Biological and Environmental Sciences and Engineering Division (BESE), Thuwal, Saudi Arabia; b King Abdullah University of Science and Technology (KAUST), Computational Bioscience Research Center (CBRC), Thuwal, Saudi Arabia; c University of Jordan, Department of Agriculture, Amman, Jordan

## Abstract

*Pseudomonas punonensis* strain D1-6 was isolated from roots of the desert plant *Erodium hirtum*, near the Dead Sea in Jordan. The genome of strain D1-6 reveals several key plant growth–promoting and herbicide-resistance genes, indicating a possible specialized role for this endophyte.

## GENOME ANNOUNCEMENT

Herbicides are chemical compounds that are used in agriculture to fight off weeds and invading species of other noncrop plants. Recent discoveries have indicated that herbicides might be generated from microbial sources such as plant endophytes to protect plants from invasive and parasitic plant species (1, 2). Some of these endophytes produce antiherbicide properties that can counteract the function of commercial herbicides. Many of the species belong to the *Pseudomonas* genus (3, 4). In this study, we isolated *P. punonensis* strain D1-6 from surface-sterilized roots of the desert plant *Erodium hirtum* near the Dead Sea (31° 40.077 N; 35° 34.538E) in Jordan. Based on 16S rRNA gene analysis, strain D1-6 was closely related to *P. punonensis* (NR_109583) with 99% identity (5) and related to *P. fulva* (NR_074659) isolated from rice paddies. Genomic DNA of strain D1-6 was extracted using Qiagen’s DNeasy blood and tissue kit following the manufacturer’s protocol. The DNA library was constructed as described previously and then sequenced using paired-end reads by Illumina MiSeq (6).

MegaBLAST (7) searches of the strain D1-6 concatenated genome against the NCBI reference genome database (http://www.ncbi.nlm.nih.gov/genome) revealed that the closest relative genome belonged to *P. fulva* 12-X (CP002727) isolated from rice paddies with a query coverage of 77% and sequence similarity of 91%. We used SPAdes assembler version 3.6 with a 1-kb contig cutoff size (8) for contig assembly and then used the Indigo pipeline (9) for gene annotation and predicted open reading frames (ORFs) using FragGeneScan (10). *De novo* assembly of MiSeq reads for strain D1-6 resulted in 32 contigs with a total length of 4,534,589 bp and a mean contig size of 141,706 bp. The *N*_50_ was 322,232 bp and the *L*_50_ was reached in six contigs; the GC content of this genome was 61.4%. The annotation of *P. punonensis* strain D1-6 resulted in 3,244 ORFs, six rRNAs, 58 tRNAs, and 103 ncRNAs.

*P. punonensis* strain D1-6 produces the well-known plant growth–promoting enzyme 1-aminocyclopropane-1-carboxylate deaminase (ACC deaminase) (EC: 3.5.99.7, K01505) (11). Other plant growth–promoting enzymes such as cholesterol oxidase (EC: 1.1.3.6) provide insect resistance (12), and polygalacturonase (EC: 3.2.1.15) increases plant resistance to both fungal and bacterial pathogens via oligogalacturonide release (13). The presence of arginine decarboxylase (EC: 4.1.1.19) was shown to increase plant resistance to salinity, heat, and dehydration (14). *P. punonensis* strain D1-6 encodes for phosphinothricin acetyltransferase genes (EC: 2.3.1.183) that are responsible for herbicide resistance (15). The genome of *P. punonensis* strain D1-6 also encodes for many enzymes that have been identified as targets for the development of herbicide-resistant plants, e.g., quinate/shikimate dehydrogenase (EC: 1.1.1.282) (16), 4-hydroxyphenylpyruvate dioxygenase (EC: 1.13.11.27) (17). The finding of phosphinothricin (PPT) and other herbicide-resistance genes in *P. punonensis* strain D1-6 may indicate a possible role for this endophyte for providing the plant host with herbicide resistance.

### Accession number(s).

The genome of *Pseudomonas punonensis* D1-6 was deposited at DDBJ/EMBL/GenBank under the accession number LWHA00000000. The version described in this paper is the first version, LWHA01000000.
